# Dynamics of pseudo‐atrophy in RRMS reveals predominant gray matter compartmentalization

**DOI:** 10.1002/acn3.51302

**Published:** 2021-02-03

**Authors:** Nicola De Stefano, Antonio Giorgio, Giordano Gentile, Maria Laura Stromillo, Rosa Cortese, Claudio Gasperini, Andrea Visconti, Maria Pia Sormani, Marco Battaglini

**Affiliations:** ^1^ Department of Medicine, Surgery and Neuroscience University of Siena Siena Italy; ^2^ San Camillo‐Forlanini Hospital Rome Italy; ^3^ Medical Affairs Department Merck Serono Rome Italy; ^4^ Biostatistics Unit, Department of Health Sciences University of Genoa Genoa Italy

## Abstract

**Objective:**

To assess the dynamics of “pseudo‐atrophy,” the accelerated brain volume loss observed after initiation of anti‐inflammatory therapies, in patients with multiple sclerosis (MS).

**Methods:**

Monthly magnetic resonance imaging (MRI) data of patients from the IMPROVE clinical study (NCT00441103) comparing relapsing‐remitting MS patients treated with interferon beta‐1a (IFNβ‐1a) for 40 weeks versus those receiving placebo (16 weeks) and then IFNβ‐1a (24 weeks) were used to assess percentage of gray (PGMVC) and white matter (PWMVC) volume changes. Comparisons of PGMVC and PWMVC slopes were performed with a mixed effect linear model. In the IFNβ‐1a‐treated arm, a quadratic term was included in the model to evaluate the plateauing effect over 40 weeks.

**Results:**

Up to week 16, PGMVC was −0.14% per month in the placebo and −0.27% per month in treated patients (*P* < 0.001). Over the same period, the decrease in PWMVC was −0.067% per month in the placebo and −0.116% per month in treated patients (*P* = 0.27). Similar changes were found in the group originally randomized to placebo when starting IFNβ‐1a treatment (week 16–40, reliability analysis). In the originally treated group, over 40 weeks, the decrease in PGMVC showed a significant (*P* < 0.001) quadratic component, indicating a plateauing at week 20.

**Interpretation:**

Findings reported here add new insights into the complex mechanisms of pseudo‐atrophy and its relation to the compartmentalized inflammation occurring in the GM of MS patients. Ongoing and forthcoming clinical trials including MRI‐derived GM volume loss as an outcome measure need to account for potentially significant GM volume changes as part of the initial treatment effect.

## Introduction

Brain volume (BV), as measured by magnetic resonance imaging (MRI)‐based computational methods, has proved clinical relevance as a marker that may reflect neurodegeneration in several neurological conditions.^1^ In patients with multiple sclerosis (MS), numerous studies have consistently shown that rates of BV loss are higher than in healthy subjects^2^, ^3^ and that accelerated BV loss is present at the earliest stage of the disease and progresses throughout the entire disease course.^4^ However, while in pure neurodegenerative disorders BV loss is mostly due to the diffuse tissue damage caused by the neurodegeneration, in a complex disease such as MS additional pathological processes that include demyelination, inflammation, and microglia activation may come into play.^5^ Thus, in MS, high rates of BV loss can also arise from resolution of inflammatory edema.^4^, ^6^ In this respect, particular attention should be given to the paradoxical acceleration of BV loss in MS brain following the initiation of most of the anti‐inflammatory disease‐modifying therapies (DMTs), referred to as “pseudo‐atrophy.”^7^

The phenomenon of pseudo‐atrophy, as occurs in MS brains, has been described in several studies^8^, ^9^, ^10^, ^11^, ^12^, ^13^, ^14^, ^15^, ^16^ but is still not fully understood. It is generally assumed to be due to the resolution of inflammatory edema or, more in general, fluid shifts have been suggested as the basis for this.^7^ However, there is no direct evidence for decreased water content in the brain of MS patients^14^ and changes in the volume of inflammatory cells, particularly glial cells, cannot be excluded.^4^, ^12^, ^16^

Independently of the mechanisms being at the basis of pseudo‐atrophy, this phenomenon certainly complicates the interpretation and the clinical impact of BV measurements in MS. Since treatments of MS aim to target not only focal inflammatory lesions but also the diffuse neurodegeneration that occurs in MS brains, MRI‐based measurements of BV have been commonly used as an endpoint in many clinical trials to monitor the effects of DMTs on BV.^17^ Although most treatments have shown to significantly reduce the rate of BV loss in the long term, a short yet variable^4^, ^10^, ^12^ period following the initiation of therapy seems to be dominated by the above‐described pseudo‐atrophy effect and has led to assess the treatment effect on BV in clinical trials only from the second year on,^18^ when the pseudo‐atrophy effect is thought to be diminished or resolved.^4^, ^7^, ^18^ In this context, it might be of great relevance to establish whether pseudo‐atrophy could be localized in specific brain tissues (i.e., white matter [WM] and/or gray matter [GM]) or regions, since this could have important implication for the design of clinical trials and for the interpretation of BV changes in clinical studies. Indeed, recent studies on the topic have shown unclear results, suggesting that either WM^10^, ^11^, ^13^ or GM may be responsible for most of the pseudo‐atrophy effect observed soon after the initiation of treatment with DMTs.

Against this background, we trusted that the use of an updated version of the SIENA method, SIENA‐XL,^19^ which allows a more robust assessment of GM and WM volumes with respect to other methods, on monthly acquired MRI data could provide new insights on the complex dynamics of pseudo‐atrophy in MS. Thus, we performed here a post‐hoc analysis of the IMPROVE clinical trial on monthly acquired MRI data of patients with relapsing‐remitting (RR) MS who were treated either with interferon beta‐1a (IFNβ‐1a) for 40 weeks or with placebo for 16 weeks followed by IFNβ‐1a for the subsequent 24 weeks.

## Methods

### Population

Monthly MRI data of RRMS patients from the IMPROVE study, a multicenter phase IIIb randomized (2:1) clinical study (ClinicalTrials.gov identifier NCT00441103) comparing patients treated with 44 μg of IFNβ‐1a given subcutaneously three times per week (tiw) (*n* = 120) versus placebo (*n* = 60) for the double‐blind phase (16 weeks). All patients then received IFNβ‐1a 44 mg sc tiw, for an additional 24 weeks in the rater‐blind phase. Details on trial design and patient characteristics as well as complete results of the clinical trial are reported elsewhere.^20^, ^21^. The study protocol was approved by the Ethics Committees of each participating center, and was conducted in accordance with the Declaration of Helsinki (1996). All patients gave written informed consent.

### MRI acquisition and analysis

Conventional brain MRI scans were performed on 1.5 Tesla magnets in all patients. MRI acquisition comprised dual‐echo (TR = 2000–3000 ms, TE1 = 15–40 ms, TE2 = 60–100 ms, 1 excitation) and T1‐weighted (T1‐W) images (TR = 400–700 ms, TE = 5–25 ms, 2 excitations) that were acquired at baseline and every 4 weeks (see 20 for details on MRI scans). All the sequences had an in‐plane resolution of 1 mm and a slice thickness of 3 mm, without gap. T1‐W images were used for the BV analysis and were processed using the SIENA‐XL^19^ method in order to obtain percentage differences of whole brain volume change (PBVC), WM volume change (PWMVC), GM volume change (PGMVC), and cortical and deep GM volume changes.

In brief:

1. The voxel intensity within each lesional region on T1‐W image was replaced with that of the surrounding WM to avoid errors in GM and WM volume assessment due to the misclassification of voxels.^22^ A subject‐specific T1‐W brain mask was created by merging all the T1‐W brain masks of that subject, each obtained with an optimized procedure for the T1‐W 2D images.^23^ This subject‐specific brain mask was then reapplied to each T1‐W image to obtain the brain image at each time point.
2. Finally, a presegmentation intensity equalization step was performed. The intensity of all the T1‐W brain images of a subject was modified to impose similar histograms of pure voxels, that is, those voxels that had 100% probability of belonging to only one tissue.
3. The GM and WM volumes of each lesion‐filled intensity‐equalized T1‐W brain image were obtained by using the FAST algorithm and the changes in a given tissue volume (i.e., GM or WM) between a pair of time points were expressed as percentage changes, that is, PGMC or PWMC. Values of deep GM volumes were obtained using the FIRST algorithm.

### Statistical analysis

On the processed images we performed the following analysis.

1. *Analysis of the double‐blind phase (week 0–16)*. Comparison of the slopes of PGMVC and PWMVC over the first 16 weeks between the two treatment arms (i.e., treated vs. placebo) was performed with a mixed effect linear model including center as random effect. A treatment‐by‐time interaction term was used to assess whether the slope of changes was different according to the treatment arm.
2. *Reliability analysis*. On the arm originally randomized to placebo, the estimation of the slopes of PGMVC and PWMVC over the last 24 weeks (after the start of treatment, week 16–40) was performed with a mixed effect linear model including center as random effect.
3. *Evaluation of a plateauing effect in treated subjects*: In the IFNβ‐1a‐treated arm, a quadratic term was included in the model to evaluate, by checking the time of maximum slope, a plateauing effect over the 40 weeks of treatment.

## Results

Baseline demographic, clinical, and MRI lesion characteristics of the study patient population are reported in Table 1. From the original patient population of 180 RRMS patients, data of 169 patients (56 originally in the placebo arm and 113 in the IFNβ‐1a‐treated arm) were available for this post‐hoc analysis. Of the total number of available scans (*n* = 1691), 266 scans (15%) were excluded due to poor scan quality. The final analysis therefore included 483 scans in the placebo arm and 942 in the IFNβ‐1a‐treated arm.

**Table 1:** Baseline demographic, clinical, and MRI lesion characteristics of the study population.

	Placebo (*n* = 60)	IFNβ‐1a (*n* = 120)
Age (years), mean ± SD	35.2 ± 10.5	34 ± 7.8
Sex, *n* (%)	M: 18 (30) F: 42 (70)	M: 32 (26.7) F: 88 (73.3)
Expanded Disability Status Scale (EDSS), median (range)	2.25 (0–5.5)	2.50 (0–5.5)
Number of T2 lesions, mean ± SD	32.60 ± 19.13	29.18 ± 15.63
T2‐lesion volume (cm^3^), mean ± SD	15.54 ± 15.73	13.47 ± 11.91
Number of T1‐Gd+ lesions, mean ± SD	3.02 ± 4.64	2.34 ± 4.28
T1‐Gd+ lesion volume (cm^3^), mean ± SD	0.43 ± 0.91	0.30 ± 0.79

No statistical differences between placebo and treated groups.

### Double‐blind phase (week 0–16)

**Figure 1:**
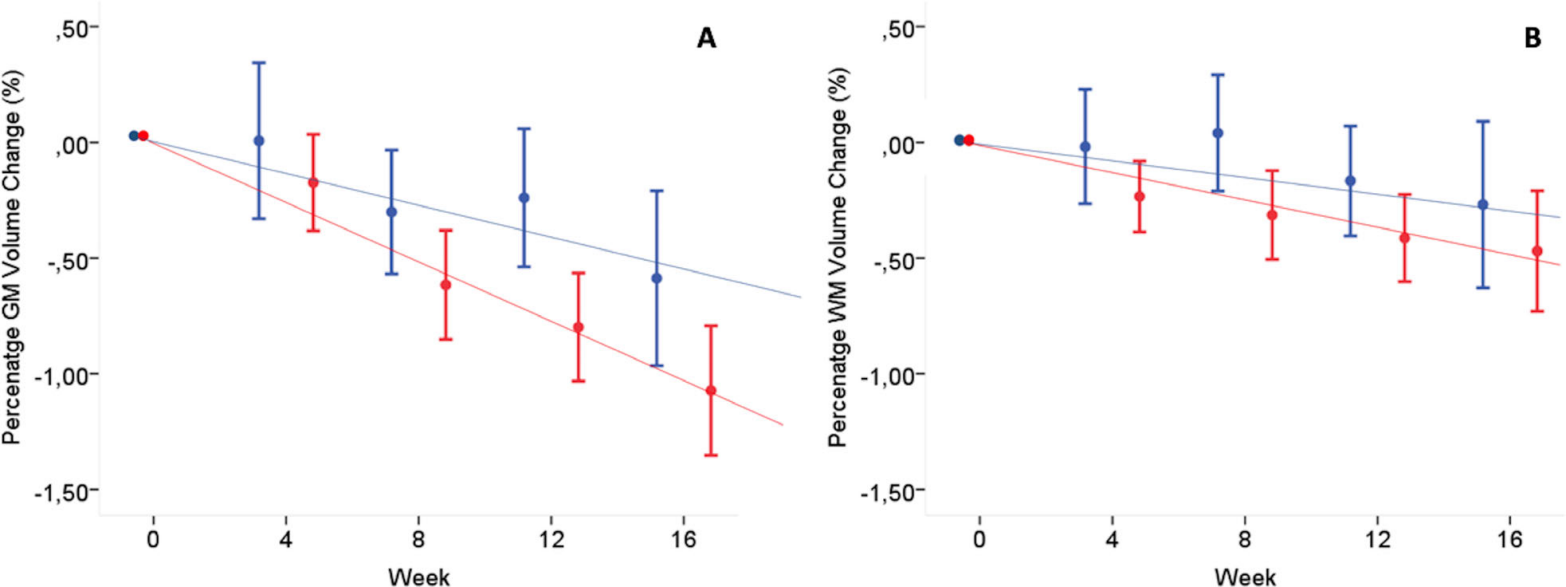
Graph showing the slope of PGMVC and PWMVC during the double‐blind period (week 0–16) in the two treatment arms. (A) Decrease in PGMVC of −0.14 ± 0.04% per month in the placebo group (blue color) and significantly more rapid decrease in the treated group (red color, −0.27 ± 0.03% per month; *P* < 0.001). (B) Decrease in PWMVC of −0.067 ± 0.03% per month in the placebo group (blue color) and slightly more pronounced decrease in the treated group (red color, −0.116 ± 0.02% per month; *P* = 0.27). See text for abbreviations. PGMVC, percent gray matter volume change; PWMVC, percent white matter volume change; IFNβ‐1a, interferon beta‐1a.

Up to week 16, the mean decrease in total BV (± standard error [SE]) was −0.10 ± 0.02% per month in the placebo group and significantly more rapid in the treated group (−0.19 ± 0.02% per month; *P* = 0.004; see Table 2). Over the same period, the decrease in PWMVC was −0.067 ± 0.03% per month in the placebo group and slightly more pronounced in the treated group (−0.116 ± 0.02% per month; *P* = 0.27). The decrease in PGMVC was −0.14 ± 0.04% per month in the placebo group and significantly more rapid in the treated group (−0.27 ± 0.03%, per month; *P* < 0.001) (Fig. 1). This rapid decrease in GM volume was concentrated in the cortical GM (placebo group: −0.13 ± 0.05%; treated group: −0.27 ± 0.04%, *P* = 0.02) rather than in the deep GM (placebo group: −0.27 ± 0.09%; treated group: −0.30 ± 0.08%, *P* = 0.92, see Table 2). In the treated arm, when the subgroups of patients with gadolinium‐enhancing (Gd+) (number of patients: 70/113 [62%], mean [±SD] number of lesions: 2.6 [±4.4]) or without (Gd−) lesions at baseline were considered, no differences were found in PGMVC after 16 weeks (−0.28 ± 0.04% vs. −0.26 ± 0.04%, *P* = 0.7) while a nonsignificant trend was seen in PWMVC over the same period (−0.15 ± 0.03% vs. −0.08 ± 0.04%, *P* = 0.1).

**Table 2:** Decrease in total and tissue‐type brain volumes during the double‐blind phase (week 0–16) in the placebo and treated groups.

	Placebo (*n* = 60)	IFNβ‐1a (*n* = 120)	*P*‐value
PBVC (%)/month, mean ± SE	−0.10 ± 0.02	−0.19 ± 0.02	0.004
PWMVC (%)/month, mean ± SE	−0.067 ± 0.03	−0.116 ± 0.02	0.27
PGMVC (%)/month, mean ± SE	−0.14 ± 0.04	−0.27 ± 0.03	<0.001
PCGMVC (%)/month, mean ± SE	−0.13 ± 0.05	−0.27 ± 0.04	0.02
PDGMVC (%)/month, mean ± SE	0.27 ± 0.09	0.30 ± 0.08	0.92

PBVC, percent brain volume change; PWMVC, percent white matter volume change; PGMVC, percent gray matter volume change; PCGMVC, percent cortical gray matter volume change; PDGMVC, percent deep gray matter volume change; SE, standard error.

### Reliability analysis

**Figure 2:**
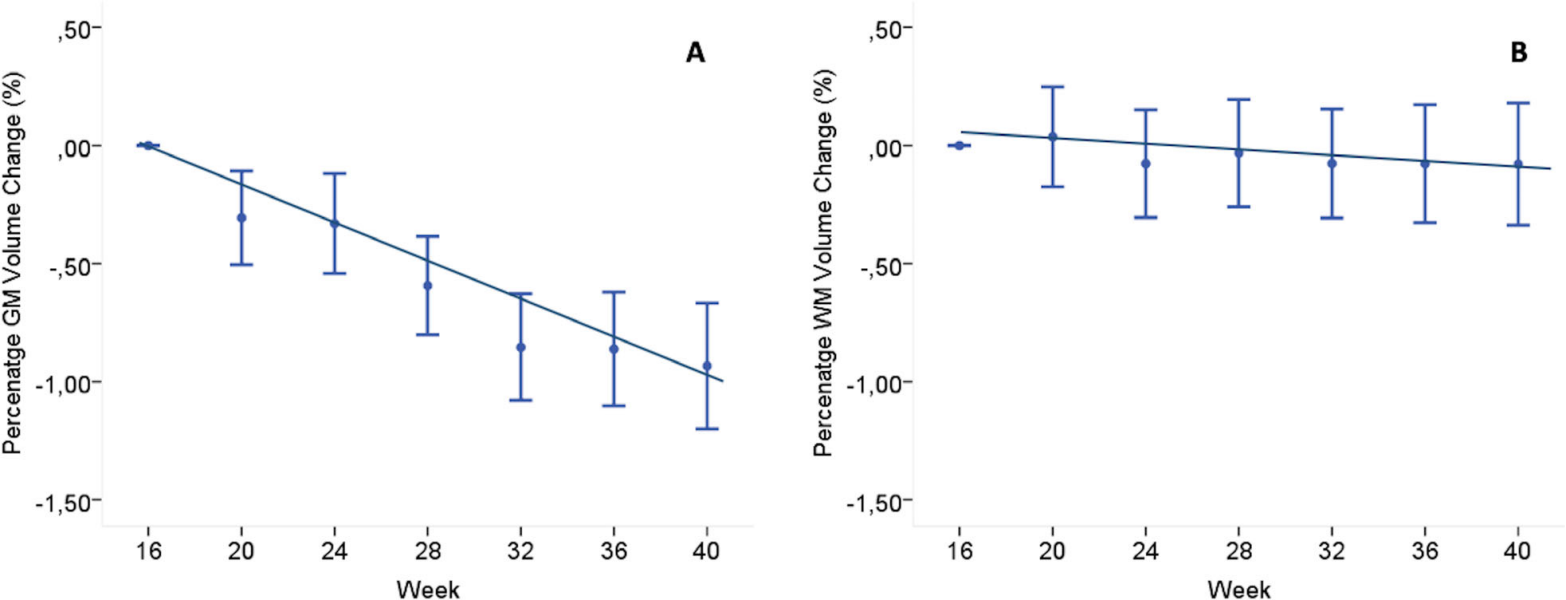
Graph showing the slope of PGMVC and PWMVC over the week 16–40 in the group originally randomized to placebo and starting treatment with IFNβ‐1a at week 16. (A) Decrease in PGMVC (−0.24 ± 0.03% per month), which was (B) more pronounced than in PWMVC (−0.016 ± 0.024%, per month). See text for abbreviations. PGMVC, percent gray matter volume change; PWMVC, percent white matter volume change; IFNβ‐1a, interferon beta‐1a.

The group originally randomized to placebo and starting treatment with IFNβ‐1a at week 16 showed, between weeks 16 and 40, more pronounced decreases in PGMVC than in PWMVC (PGMVC week 16–40 = −0.24 ± 0.03% per month, PWMVC week 16–40 = −0.016 ± 0.024% per month) (Fig. 2). This confirmed the presence of more pronounced changes in the GM volume as shown in the group of patients treated since the beginning.

### Plateauing effect

**Figure 3:**
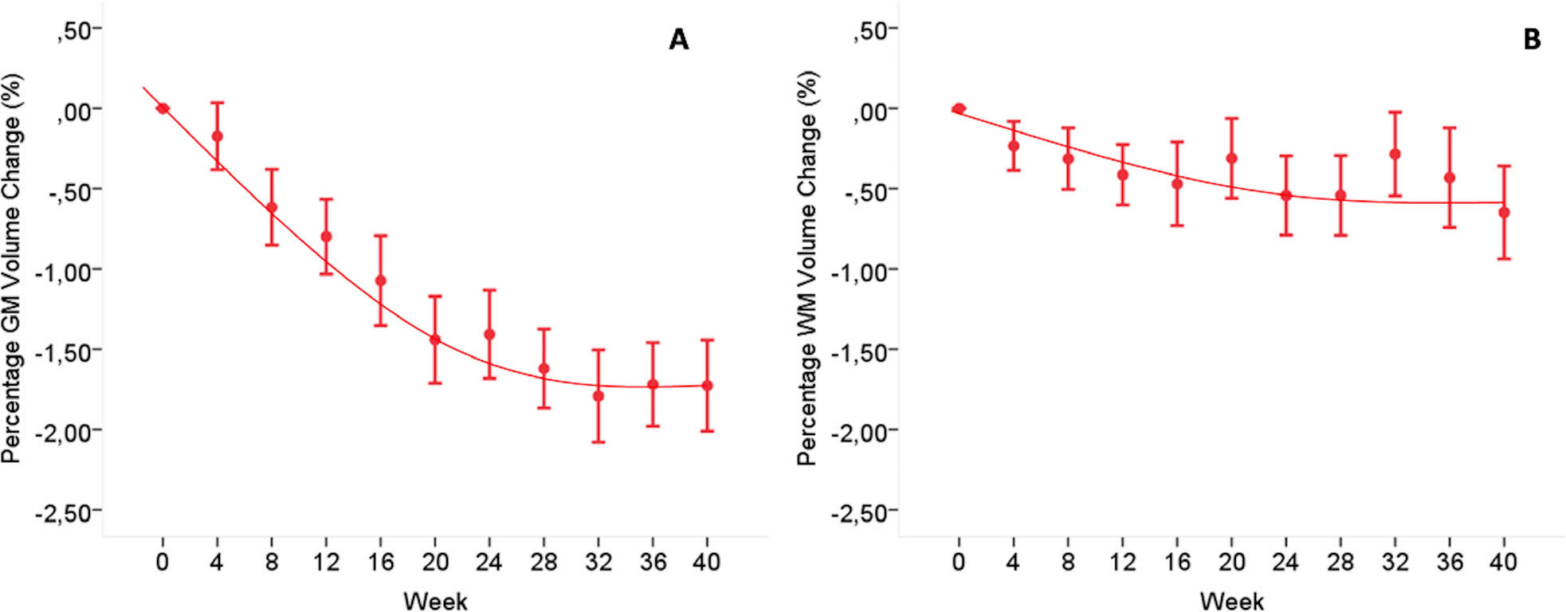
Graph showing the 40‐week slope of PGMVC and PWMVC in the originally IFNβ‐1a‐treated groups. (A) Decreasein PGMVC with a significant (*P* < 0.001) quadratic component, indicating a plateauing that was estimated to start at week 20. (B) Over the same time period, it is shown the small decrease in PWMVC with only a trend (*P* = 0.06) for a quadratic slope with a plateau also starting at week 20. See text for abbreviations. PGMVC, percent gray matter volume change; PWMVC, percent white matter volume change; IFNβ‐1a, interferon beta‐1a.

In the treated group, over 40 weeks the decrease in PGMVC showed a significant (*P* < 0.001) quadratic component, indicating a plateauing of the PGMVC that was estimated to start at week 20. Over the same time period, the small decreases in PWMVC showed only a trend (*P* = 0.06) for a quadratic slope with a plateau also starting at week 20 (Fig. 3).

## Discussion

In monthly acquired MRI data, RRMS patients treated with IFNβ‐1a showed a more pronounced pseudo‐atrophy effect in GM than in WM, with a maximum decrease up to week 20 in both brain tissues. A similar dynamics was seen in patients originally treated with placebo when they started therapy with IFNβ‐1a (reliability analysis). These results are in contradiction of the general understanding that pseudo‐atrophy, as expression of the resolution of inflammation (i.e., edema), should be mostly or exclusively prominent in the WM compartment in MS. They suggest, instead, that GM needs to be considered to factor out the confounding effects of anti‐inflammatory drugs when measuring BV changes soon after therapy initiation.

In several MS clinical trials, negative results were, at least in part, attributed to a pseudo‐atrophy effect, and interpreted as caused by the link of BV measurements to the presumed treatment‐associated resolution of inflammatory activity and edema.^4^ In line with this, in some trials^4^, ^17^, ^18^, ^24^ significant treatment effects were observed only after the first year of treatment, when the pseudo‐atrophy effect is presumed to be minor or over.^4^, ^18^ It must be stressed, however, that the dynamics of pseudo‐atrophy are still largely unknown. In this context, previous results have shown contradictory results in relation to the contribution of WM and GM compartments to pseudo‐atrophy. In some observational studies, WM appeared to contribute most to the observed pseudo‐atrophy effect in patients treated with IFNβ‐1a^13^ or natalizumab.^10^ However, another previous study on 84 patients treated with IFNβ‐1a^11^ showed that, while higher baseline number of Gd+ lesions was predictive of larger decreases in whole brain and WM volumes, mean volume changes in the first year of treatment were definitely more pronounced in the GM (−0.79%) than in WM (−0.11%). In addition, another small observational study on 23 RRMS patients treated with IFNβ‐1a showed most of the short‐term (i.e., 3 months) treatment‐induced pseudo‐atrophy effect in the GM compartment.^15^ Finally, a study on a small group (*n* = 19) of MS patients who underwent immunoablation and autologous hematopoietic stem cell transplantation observed that, in these highly active patients, the average volume loss during the first month of follow‐up was significantly greater in the GM (−2.12%) than in the WM (−0.96%).^14^

Taken together, our and previous data suggest that the presence of mechanisms of pseudo‐atrophy can affect both GM and WM tissues. However, GM volume changes appear somehow prevalent when data are analyzed within one or few months after therapy start.^14^, ^15^ In this context, data presented here should be particularly convincing. First, we analyzed a unique MRI dataset of RRMS patients, acquired every 4 weeks, who were randomized to the start of IFNβ‐1a therapy (for 40 weeks) or placebo (for 16 weeks). This allowed an earlier and more detailed analysis, in comparison with previous studies, of differences in the dynamics of GM and WM volume changes occurring after the start of IFNβ‐1a therapy. It also showed that volume decreases with similar slopes for both brain compartments (up to 20 weeks), but with a magnitude much more pronounced in the GM than in WM. Second, in our reliability analysis, we had the opportunity to repeat the same analysis on the group of patients previously randomized to placebo when they started IFNβ‐1a therapy (for 24 weeks). Interestingly, data largely confirmed the previous analysis, clearly showing a more pronounced effect in the GM than in WM volume changes. Finally, it must be stressed here that ours and previous data showing pseudo‐atrophy effect in the GM have been analyzed with a new generation of imaging processing methods (i.e., SIENA‐XL,^19^ SIENAX Multi Time Points,^25^ Jacobian integration method^26^) using less biased and more precise estimates for GM volumes that are able to overcome some of the previous technical limitations in this measurement.^19^, ^27^

The pathophysiological mechanisms responsible for pseudo‐atrophy are still unclear. It is generally believed that BV may decrease after anti‐inflammatory therapy, independently of the actual tissue loss, because of the loss of tissue water that would accompany the resolution of inflammatory activity.^4^, ^6^ In line with this, previous studies have shown a close relationship between the short‐term rapid decrease in BV found after the initiation of an anti‐inflammatory therapy and the amount of inflammation (i.e., number of Gd+ lesions) present at therapy start.^4^, ^10^, ^11^ This was somehow confirmed in our short‐term study, where patients who were Gd+ at baseline showed a trend for more rapid WM volume changes in comparison to Gd− patients after only 16 weeks. This implies that mechanisms of pseudo‐atrophy affect mainly WM, likely due to the resolution of inflammatory activity compartmentalized near the active WM lesions. It is interesting to note that in patients with highly inflammatory activity who underwent immunoablation and autologous hematopoietic stem cell transplantation, Lee and co‐workers^12^ have excluded effects on pseudo‐atrophy due to tissue water fluctuation after measuring changes in tissue water content with a two‐point estimated “pseudo” T2‐relaxation time.^28^ It is therefore unlikely that mechanisms underlying pseudo‐atrophy are simply caused by the fluid shift in following the administration of an anti‐inflammatory drug. Indeed, decrease in the volume of inflammatory cells, such as activated microglia and macrophages, which would be indistinguishable from true tissue loss on MRI, may have a relevant role on this.^12^, ^29^

In our study, GM (rather than WM) was the brain compartment showing most of the pseudo‐atrophy in RRMS patients soon after the initiation of IFNβ‐1a. This was seen in both the IFNβ‐1a arm and in the arm originally treated with placebo, when patients started therapy with IFNβ‐1a. While in the WM, particularly at early stages, the inflammation is mainly related to the focal, perivascular invasion of T and B lymphocytes leading to the formation of the classical active demyelinated plaques, another type of more diffuse inflammation seems to be prominent in the GM.^30^ This accumulates in the large connective tissue spaces of the brain and spinal cord, prominently affecting the meninges and the large perivascular Virchow‐Robin spaces, and leads to tissue injury that is, at least in part, mediated by a cascade involving microglia and macrophage activation, oxidative stress, and mitochondrial damage. This response may chronically generate inflammatory, cytotoxic, and possibly myelinotoxic mediators that, by circulating within the cerebrospinal fluid, may diffuse freely throughout the subarachnoid space and, by crossing the pial membrane toward the adjacent GM, mediate the extensive subpial GM injury in MS.^30^, ^31^ In line with this, a “surface‐in” gradient of tissue damage has been observed in both cortical^32^ and subcortical^33^ GM, strongly supportive of the diffusion of cytotoxic soluble factors from the meninges.^34^ Within this framework, the prominent GM volume decrease found in our study in MS patients soon after starting IFNβ‐1a should not be generically interpreted as a decrease in water content, but as the expression of a potentially beneficial anti‐inflammatory effect of the drug and more evident in our study in the cortical GM compartment (see Table 2). This would lead to a diffuse decrease in the activated microglia and macrophages and the consequent reduction in the soluble neurotoxic factors diffusing from the meningeal compartment and contributing to GM damage and the consequent worsening in clinical disability.^30^, ^32^ Further imaging and neuropathological studies are necessary to support this.

While data on pseudo‐atrophy remain discordant and further research is warranted to clarify the contribution of GM atrophy to this interesting phenomenon, evidence suggests that it cannot be interpreted as a simple resolution of inflammatory edema.^9^, ^12^, ^16^, ^35^ Findings reported here add new insights into the complex mechanisms of pseudo‐atrophy and its relation to the compartmentalized inflammation occurring in the GM of MS patients. Whether this is an expression of a beneficial anti‐inflammatory effect (i.e., decrease in soluble neurotoxic factors) of the drug or, as recently shown in MS patients after immunoablative autologous hematopoietic stem cell transplantation,^35^ of a transient neurotoxicity following the intense chemotherapy, needs to be demonstrated. In either case, ongoing and forthcoming clinical trials including MRI‐derived GM volume loss as an outcome measure need to account for potentially significant GM volume changes as part of the initial treatment effect.

## Conflict of Interests

NDS is a consultant for Biogen, Merck KGaA, Novartis, Roche, Sanofi‐Genzyme, Schering and Teva; has grants or grants pending from FISM and Novartis, is on the speaker bureaus of Biogen, Teva, Novartis, Sanofi‐Genzyme, Roche, and Merck KGaA; has received travel funds from Merck KGaA, Novartis, Roche, Sanofi‐Genzyme and Teva. RC was awarded 2019 MAGNIMS‐ECTRIMS fellowship. CG has received fee as speaker or advisory board by Teva, Novartis, Roche, Merck KGaA, Bayer, Almirall, Biogen. AV and AP are employees of Merck Serono S.p.A., Rome, Italy, an affiliate of Merck KGaA, Darmstadt, Germany. MPS has received consulting fees from Biogen, GeNeuro, Genzyme, MedDay, Merck KGaA, Novartis, Roche and Teva. AG, GG, MLS, and MB have nothing to disclose.

## Author Contributions

NDS: Conceptualization, Methodology, Investigation, Resources, Writing Original Draft, Supervision, Funding acquisition. AG, MLS, and RC: Formal analysis, Investigation, Writing – Review & Editing. GG: Software, Validation, Formal analysis, Writing – Review & Editing. CG: Investigation, Resources, Writing – Review & Editing. AV & AP: Resources, Writing – Review & Editing. MPS: Methodology, Formal analysis, Writing – Review & Editing. MB: Methodology, Software, Validation, Formal analysis, Writing – Review & Editing, Visualization.

## Data Availability

The dataset used and analyzed in the current study is available from the corresponding author upon reasonable request.
